# Supplementary material for: Following Evidence-Based Recommendations for Perioperative Pain Management after Cesarean Section Is Associated with Better Pain-Related Outcomes: Analysis of Registry Data
Source: J Clin Med. 2023 Jan 14;12(2):676. doi: 10.3390/jcm12020676 (PMC9864952; doi:10.3390/jcm12020676)
Supplement: Supplementary file 1 [file jcm-12-00676-s001.zip › jcm-2050866-supplementary S1.pdf]

SUPPLEMENT I

Contents

**S-I-Table S1**	Sources that formed the basis for selecting the elements of care – as described in Section 2.3 of the manuscript	2
**S-I-Table S2**	Single doses and maximum daily doses for non-opioid medications<br>Example of calculating the "full daily dose" of non-opioid analgesics	3
**S-I-Table S3**	Countries from which women were surveyed	4
**S-I-Table S4**	Details on the type of anesthesia in the cohort	5
**S-I-Table S5**	Descriptive statistics of the patient-reported outcomes for the 'Care' groups in the total cohort	6
**References**		8

S-I-Table S1 Sources which formed the basis for selecting the elements of care – as described in Section 2.3 of the manuscript

**Intervention / Source**	**Sutton & Carvalho [1]**	**PROSEPCT [2]**	***Acute Pain Management: Scientific Evidence* [3]**	**Chou et al [4]**	**Royal College of Anaesthetists [5]**	**NICE [6]**	**ASA Task Force on Obstetric Anesthesia [7]**	***Grape et al [8]***	**Bamigboye & Hofmeyr *[9]***
**Pre / intra-operative**									
Regional (spinal or epidural) – opioid, any				x			x		
Regional (spinal or epidural) – long acting opioid	x	x	x		x	x			
General anesthesia (GA): wound infiltration / TAP	x		x	x				x	x
GA addressed						x			
Paracetamol & NSAID intra-op	x	X	x			x			
**After surgery**									
NSAID					x				
Paracetamol & NSAID	x	x	x	x		x			
Opioid, as rescue	x	x	x	x	x	x			
Pain assessment	x	x	x	x	x	x			

These are references published before or during the study's time period. Clinicians taking part in the study could have used these as references.

S-I-Table S2. Doses used to calculate sufficient single and cumulative daily doses [10,11]

drug	daily dose [mg]	single dose [mg]
celecoxib	200	200
clonixin	600	200
dexketoprofen	150	50
diclofenac	150	75
etoricoxib	120	60
flurbiprofen	300	50
ibuprofen	2400	400
indometacin	150	50
ketoprofen	150	50
ketorolac	150	30
mefenamic acid	1250	500
metamizol	4000	500
naproxen	1000	500
nefopam	90	30
paracetamol	4000	500
parecoxib	80	40

Example of calculating the "full daily dose" of non-opioid analgesics:

	patient received	daily dose	% of daily dose
intraoperative	50 mg ketoprofen	150 mg	33 %
	1000 mg paracetamol	4000 mg	25 %
PACU	50 mg indometacine	150 mg	33 %
normal ward	2000 mg paracetamol	4000 mg	50 %
**total:**			**141 %**

start of surgery	01.01.2019 08:15
time of survey	02.01.2019 14:30
Δ time	30.25 hours
**Δ time (in % of days)**	**126%** (i.e. 30.25/24)

The total percentage of daily doses (141%) is larger compared to the "standardized" time between start of surgery until time of survey (126%). Thus, the treatment element was regarded as fulfilled.

S-I-Table-S3. Countries from which women were su rveyed:

		**number of wards**
**High income countries:**	Switzerland	5
	Germany	2
	Israel	2
	France	1
	Belgium	1
	Saudi-Arabia	1
	China: Hong Kong	1
**Middle and low income countries:**	China	2
	South Africa	2
	Serbia	1
	Colombia	1
	Malaysia	1
	Philippines	1
	Indonesia	1
	Kosovo	1
	Rwanda	1

S-I-Table S4. Anaesthesia techniques employed in the care groups

		regional anaesthesia group		combined group		all women with regional anaesthesia	
		n = 4,428		n = 113		n = 4,541	
**Type**	**Specification**	**N**	**%**	**n**	**%**	**n**	**%**
**Spinal**	**Only**	3,879	87.6	63	55.8	3,942	86.8
	**including others and TAP**	3,883	87.7	64	56.6	3,947	86.9
**Epidural**	**Only**	386	8.7	47	41.6	433	9.5
	**including others and TAP**	387	8.7	47	41.6	434	9.6
**Spinal and epidural**	**Only**	158	3.6	1	0.9	159	3.5
	**including TAP**	158	3.6	2	1.8	160	3.5
**Spinal**	**any combination**	4,041	91.3	66	58.4	4,107	90.4
**Epidural**	**any combination**	545	12.3	49	43.4	594	13.1

S-I-Table S5. Descriptive statistics of patient reported outcomes **for the 'Care' groups in the total cohort (n = 5,182)**. Results are shown for women in the 'Incomplete' care group ('Incomplete', n = 3,251), women with intra-operative care only (Intra-operative, n = 190), women with postoperative care only (Post-operative, n = 722) and women with 'Full' care (Full, n = 1,019).

variable	protocol group	n	%	95% CI	
**worst pain intensity ≥ 7/10 NRS**	Incomplete	1,950	60.0	58.3	61.7
	Intra-operative	102	53.7	46.3	60.9
	Post-operative	411	56.9	53.2	60.6
	Full	505	49.6	46.4	52.7
**time in severe pain ≥ 50 %**	Incomplete	1,745	53.7	51.9	55.4
	Intra-operative	81	42.6	35.5	50.0
	Post-operative	232	32.1	28.7	35.7
	Full	280	27.5	24.8	30.3
**interference: in bed ≥ 6/10 NRS**	Incomplete	1,809	55.6	53.9	57.4
	Intra-operative	108	56.8	49.5	64.0
	Post-operative	462	64.0	60.4	67.5
	Full	508	49.9	46.7	53.0
**interference: breathing/coughing ≥ 5/10 NRS**	Incomplete	1,650	50.8	49.0	52.5
	Intra-operative	99	52.1	44.8	59.4
	Post-operative	424	58.7	55.0	62.3
	Full	534	52.4	49.3	55.5
**interference: sleep ≥ 5/10 NRS**	Incomplete	1,512	46.5	44.8	48.2
	Intra-operative	81	42.6	35.5	50.0
	Post-operative	306	42.4	38.7	46.1
	Full	362	35.5	32.6	38.6
**anxiety ≥ 4/10 NRS**	Incomplete	1,615	49.7	47.9	51.4
	Intra-operative	75	39.5	32.5	46.8
	Post-operative	274	38.0	34.4	41.6
	Full	282	27.7	24.9	30.5
**helplessness ≥ 4/10 NRS**	Incomplete	1,620	49.8	48.1	51.6
	Intra-operative	67	35.3	28.5	42.5
	Post-operative	292	40.4	36.8	44.1
	Full	248	24.3	21.7	27.1
**dizziness ≥ 4/10 NRS**	Incomplete	1,144	35.2	33.5	36.9
	Intra-operative	57	30.0	23.6	37.1
	Post-operative	199	27.6	24.3	31.0
	Full	272	26.7	24.0	29.5
**drowsiness ≥ 5/10 NRS**	Incomplete	928	28.5	27.0	30.1
	Intra-operative	61	32.1	25.5	39.2
	Post-operative	186	25.8	22.6	29.1
	Full	289	28.4	25.6	31.2
**itch ≥ 3/10 NRS**	Incomplete	720	22.1	20.7	23.6
	Intra-operative	71	37.4	30.5	44.7
	Post-operative	212	29.4	26.1	32.8
	Full	377	37.0	34.0	40.0
**nausea ≥ 4/10 NRS**	Incomplete	688	21.2	19.8	22.6
	Intra-operative	25	13.2	8.7	18.8
	Post-operative	142	19.7	16.8	22.8
	Full	133	13.1	11.0	15.3

variable	protocol group	n	%	95% CI	
**satisfaction ≤ 6/10 NRS**	Incomplete	1,124	34.6	32.9	36.2
	Intra-operative	36	18.9	13.6	25.3
	Post-operative	122	16.9	14.2	19.8
	Full	141	13.8	11.8	16.1
**Would have liked to receive more treatment**	Incomplete	1,494	46.0	44.2	47.7
	Intra-operative	62	32.6	26.0	39.8
	Post-operative	162	22.4	19.4	25.7
	Full	255	25.0	22.4	27.8

References

1. Sutton CD, Carvalho B Optimal Pain Management After Cesarean Delivery. *Anesthesiology Clinics* 2017; **35:** 107-24.

2. C-Section. Better Postoperative Pain Management (prospect). Available online: https://archive.postoppain.org/ (accessed on 20, December, 2020).

3. Schug SA, Palmer GM, Scott DA, et al. *Acute Pain Management: Scientific Evidence (5th edition), ANZCA & FPM*,, 5th edn. Melbourne, ANZCA & FPM, 2020.

4. Chou R, Gordon DB, de Leon-Casasola OA, et al. Management of Postoperative Pain: A Clinical Practice Guideline From the American Pain Society, the American Society of Regional Anesthesia and Pain Medicine, and the American Society of Anesthesiologists' Committee on Regional Anesthesia, Executive Committee, and Administrative Council. *Journal of Pain* 2016; **17:** 131-57.

5. Colvin, J.R., Pedon, C.J., (Eds.) Raising the Standard: A compendium of Audit Recipes for Continuous Quality Improvement in Anaesthesia; The Royal College of Anaesthetists: London, UK, 2012.

6. National Collaborating Centre for Women's and Children's Health. *Caesarean Section, NICE Clinical Guideline [CG132]*; Royal College of Obstetricians and Gynaecologists: London, UK, 2011..

7. Practice Guidelines for Obstetric Anesthesia: An Updated Report by the American Society of Anesthesiologists Task Force on Obstetric Anesthesia and the Society for Obstetric Anesthesia and Perinatology. *Anesthesiology* 2016; **124:** 270-300.

8. Grape S, Kirkham KR, Albrecht E Transversus abdominis plane block versus local anaesthetic wound infiltration for analgesia after caesarean section: A systematic review and meta-analysis with trial sequential analysis. *European Journal of Anaesthesiology* 2021.

9. Bamigboye AA, Hofmeyr GJ Local anaesthetic wound infiltration and abdominal nerves block during caesarean section for postoperative pain relief. *Cochrane Database Syst Rev* 2009**:** Cd006954.

10. Principles of Analgesic Use in the Treatment of Acute Pain and Chronic Cancer Pain, 4th ed.; Clinical Pharmacy; American Pain Society: Chicago, IL, USA, 1999.

11. Meissner, W. *Akutschmerz Taschenbuch (Acute Pain Pocket Book)*; Medizinisch Wissenschaftliche Verlagsgesellschaft: Berlin, Germany, 2014
