# Supplementary material for: Following Evidence-Based Recommendations for Perioperative Pain Management after Cesarean Section Is Associated with Better Pain-Related Outcomes: Analysis of Registry Data
Source: J Clin Med. 2023 Jan 14;12(2):676. doi: 10.3390/jcm12020676 (PMC9864952; doi:10.3390/jcm12020676)
Supplement: Supplementary file 1 [file jcm-12-00676-s001.zip › jcm-2050866-supplementary S2.pdf]

SUPPLEMENT II

Contents

1 Descriptive statistics for the pain composite scores 2

2 Model details: Total cohort
2.1 Pain Composite Score: total 3
2.2 Pain Composite Score: intensity sub-score 4
2.3 Pain Composite Score: interference sub-score 5
2.4 Pain Composite Score: side effects sub-score 6

3 Sensitivity analysis for the total Pain Composite Score 7

4 Model details: Regional anaesthesia
4.1 Pain Composite Score: total 9
4.2 Pain Composite Score: intensity sub-score 10
4.3 Pain Composite Score: interference sub-score 11
4.4 Pain Composite Score: side effects sub-score 12

1 Descriptive statistics for the pain composite scores

Descriptive statistics (mean and standard deviation, SD) of the pain composite scores (PCS) and sub-scores for pain intensity ($PCS_{intensity}$), pain interference ($PCS_{interference}$) and side effects (PCS_{side}). Results are shown separately for the total cohort and women with regional anaesthesia (no general anaesthesia). In addition, the patient numbers (n) are shown.

		Total cohort			Regional anaesthesia		
**Variable**	**Care group**	**Mean**	**SD**	**n**	**Mean**	**SD**	**n**
**PCS_{total}**	Incomplete	3.82	1.74	3,071	3.92	1.77	2,509
	Intra-operative	3.52	1.66	186	3.55	1.65	167
	Post-operative	3.49	1.58	715	3.51	1.56	603
	Full care	3.25	1.54	1,010	3.19	1.52	934
**$PCS_{intensity}$**	Incomplete	5.02	2.05	3,071	5.19	2.09	2,509
	Intra-operative	4.39	2.04	186	4.37	2.04	167
	Post-operative	4.01	1.77	715	3.99	1.72	603
	Full care	3.98	1.94	1,010	3.88	1.88	934
**$PCS_{interference}$**	Incomplete	4.42	2.36	3,071	4.54	2.40	2,509
	Intra-operative	4.05	2.30	186	4.08	2.35	167
	Post-operative	4.25	2.09	715	4.27	2.05	603
	Full care	3.65	2.03	1,010	3.58	2.01	934
**PCS_{side}**	Incomplete	2.16	2.10	3,071	2.20	2.17	2,509
	Intra-operative	2.20	2.06	186	2.27	2.05	167
	Post-operative	2.14	1.98	715	2.21	2.02	603
	Full care	2.19	1.96	1,010	2.19	1.96	934

2 Model details: Total cohort

2.1 Pain Composite Score: total

Model parameter for the **total pain composite score** ($PCS_{:totall}$, n = 4,982 patients). The PCS_{total} was z-standardized before modelling. Significant regression coefficients are printed in bold (coefficient of determination: $R^2_{marginal}$= 0.025, $R^2_{conditional}$= 0.216).

Variable	[Reference/Unit]	β	95%CI		p
(Intercept)		-0.27	-0.59	0.06	0.114
**Perioperative treatment protocol:**					
Intra-operative	[*vs.* Incomplete]	-0.27	-0.42	-0.11	**<0.001**
Post-operative	[*vs.* Incomplete]	-0.07	-0.19	0.04	0.216
Full care	[*vs.* Incomplete]	-0.36	-0.49	-0.23	**<0.001**
**Group:**					
General anaesthesia (GA)	[*vs.* RA, regional anaesthesia]	0.16	0.04	0.28	**0.009**
Combined GA + RA	[*vs.* RA]	0.04	-0.14	0.22	0.659
Age	[years]	0.00	-0.01	0.00	0.138
Pre-existing chronic pain	[*vs.* no]	0.21	0.11	0.31	**<0.001**
Ward: any opioid	[*vs.* none]	0.17	0.10	0.25	**<0.001**
Income level: high	[*vs.* others]	0.32	-0.07	0.70	0.120

Estimated marginal means (covariate adjusted means) and 95% confidence intervals of the $\mathbf{PCS_{total}}$ (n = 4,982 patients). Results are shown for the peri-operative treatment protocol groups. The PCS_{total} was z-standardized before modelling.

Care group	mean	95% CI	
Incomplete	0.04	-0.17	0.25
Intra-operative	-0.23	-0.47	0.02
Post-operative	-0.03	-0.25	0.18
Full care	-0.32	-0.54	-0.09

Contrasts (differences of the estimated marginal means) and 95% confidence intervals for the **total pain composite score** (PCS_{total}, n = 4,982 patients). Significant contrasts ($p < 0.05$) are printed in bold. The PCS_{total} was z-standardized before modelling. Thus, differences can be interpreted as differences in standard deviations.

contrast			estimate	95% CI		p
Incomplete	*vs.*	Intra-operative	-0.27	-0.42	-0.11	**0.002**
Incomplete	*vs.*	Post-operative	-0.07	-0.19	0.04	0.432
Incomplete	*vs.*	Full care	-0.36	-0.49	-0.23	**<0.001**
Intra-operative	*vs.*	Post-operative	0.19	0.03	0.36	0.064
Intra-operative	*vs.*	Full care	-0.09	-0.25	0.06	0.432
Post-operative	*vs.*	Full care	-0.28	-0.40	-0.17	**<0.001**

2.2 Pain Composite Score: intensity sub-score

Model parameter for the **pain composite intensity sub-score** ($PCS_{intensity}$, n = 4,982 patients). The $PCS_{intensity}$ was z-standardized before modelling. Significant regression coefficients are printed in bold (coefficient of determination: $R^2_{marginal}$= 0.025, $R^2_{conditional}$= 0.249).

Variable	[Reference/Unit]	β	95%CI		p
(Intercept)		-0.45	-0.78	-0.11	**0.013**
**Perioperative treatment protocol:**					
Intra-operative	[*vs.* Incomplete]	-0.20	-0.35	-0.06	**0.007**
Post-operative	[*vs.* Incomplete]	-0.05	-0.16	0.07	0.428
Full care	[*vs.* Incomplete]	-0.18	-0.30	-0.05	**0.006**
**Group:**					
General anaesthesia (GA)	[*vs.* RA, regional anaesthesia]	0.16	0.04	0.27	**0.007**
Combined GA + RA	[*vs.* RA]	0.09	-0.08	0.26	0.315
Age	[years]	0.00	0.00	0.01	0.206
Pre-existing chronic pain	[*vs.* no]	0.15	0.06	0.25	**0.002**
Ward: any opioid	[*vs.* none]	0.21	0.14	0.28	**<0.001**
Income level: high	[*vs.* others]	-0.04	-0.45	0.36	0.843

Estimated marginal means (covariate adjusted means) and 95% confidence intervals of the **pain composite intensity sub-score** ($PCS_{intensity}$, n = 4,982 patients). Results are shown for the peri-operative treatment protocol groups. The $PCS_{intensity}$ was z-standardized before modelling.

group	mean	95% CI	
Incomplete	-0.11	-0.33	0.11
Intra-operative	-0.31	-0.56	-0.06
Post-operative	-0.15	-0.38	0.07
Full care	-0.29	-0.52	-0.06

Contrasts (differences of the estimated marginal means) and 95% confidence intervals for the **pain composite intensity sub-score** ($PCS_{intensity}$, n = 4,982 patients). Significant contrasts ($p < 0.05$) are printed in bold. The $PCS_{intensity}$ was z-standardized before modelling. Thus, differences can be interpreted as differences in standard deviations.

contrast			estimate	95% CI		p
Incomplete	*vs.*	Intra-operative	-0.20	-0.35	-0.06	**0.036**
Incomplete	*vs.*	Post-operative	-0.05	-0.16	0.07	0.856
Incomplete	*vs.*	Full care	-0.18	-0.30	-0.05	**0.036**
Intra-operative	*vs.*	Post-operative	0.16	0.00	0.31	0.153
Intra-operative	*vs.*	Full care	0.02	-0.13	0.17	0.856
Post-operative	*vs.*	Full care	-0.13	-0.24	-0.02	0.076

2.3 Pain Composite Score: interference sub-score

Model parameter for the **pain composite interference sub-score** ($PCS_{interference}$, n = 4,982 patients). The $PCS_{interference}$ was z-standardized before modelling. Significant regression coefficients are printed in bold (coefficient of determination: $R^2_{marginal}$= 0.025, $R^2_{conditional}$= 0.225).

Variable	[Reference/Unit]	β	95%CI		p
(Intercept)		-0.21	-0.54	0.12	0.226
**Perioperative treatment protocol:**					
Intra-operative	[*vs.* Incomplete]	-0.24	-0.39	-0.08	**0.002**
Post-operative	[*vs.* Incomplete]	-0.07	-0.19	0.05	0.240
Full care	[*vs.* Incomplete]	-0.35	-0.49	-0.22	**<0.001**
**Group:**					
General anaesthesia (GA)	[*vs.* RA, regional anaesthesia]	0.19	0.07	0.31	**0.002**
Combined GA + RA	[*vs.* RA]	0.03	-0.15	0.21	0.722
Age	[years]	0.00	-0.01	0.00	0.152
Pre-existing chronic pain	[*vs.* no]	0.10	0.00	0.20	0.052
Ward: any opioid	[*vs.* none]	0.18	0.11	0.26	**<0.001**
Income level: high	[*vs.* others]	0.38	-0.01	0.78	0.071

Estimated marginal means (covariate adjusted means) and 95% confidence intervals of the **pain composite interference sub-score** ($PCS_{interference}$, n = 4,982 patients). Results are shown for the peri-operative treatment protocol groups. The $PCS_{interference}$ was z-standardized before modelling.

group	mean	95% CI	
Incomplete	0.09	-0.13	0.31
Intra-operative	-0.15	-0.40	0.10
Post-operative	0.02	-0.20	0.24
Full care	-0.27	-0.50	-0.04

Contrasts (differences of the estimated marginal means) and 95% confidence intervals for the **pain composite interference sub-score** ($PCS_{interference}$, n = 4,982 patients). Significant contrasts ($p < 0.05$) are printed in bold. The $PCS_{interference}$ was z-standardized before modelling. Thus, differences can be interpreted as differences in standard deviations.

contrast			estimate	95% CI		p
Incomplete	*vs.*	Intra-operative	-0.24	-0.39	-0.08	**0.010**
Incomplete	*vs.*	Post-operative	-0.07	-0.19	0.05	0.297
Incomplete	*vs.*	Full care	-0.35	-0.49	-0.22	**<0.001**
Intra-operative	*vs.*	Post-operative	0.17	0.00	0.33	0.143
Intra-operative	*vs.*	Full care	-0.12	-0.28	0.04	0.297
Post-operative	*vs.*	Full care	-0.28	-0.40	-0.17	**<0.001**

2.4 Pain Composite Score: side effects sub-score

Model parameter for the **pain composite side effects sub-score** (PCS_{side}, n = 4,982 patients). The PCS_{side} was z-standardized before modelling. Significant regression coefficients are printed in bold (coefficient of determination: $R^2_{marginal}$= 0.018, $R^2_{conditional}$= 0.075).

Variable	[Reference/Unit]	β	95%CI		p
(Intercept)		-0.03	-0.26	0.20	0.804
**Perioperative treatment protocol:**					
Intra-operative	[*vs.* Incomplete]	-0.17	-0.32	-0.01	**0.038**
Post-operative	[*vs.* Incomplete]	-0.04	-0.16	0.07	0.465
Full care	[*vs.* Incomplete]	-0.23	-0.37	-0.10	**<0.001**
**Group:**					
General anaesthesia (GA)	[*vs.* RA, regional anaesthesia]	0.02	-0.11	0.14	0.787
Combined GA + RA	[*vs.* RA]	-0.01	-0.19	0.17	0.918
Age	[years]	-0.01	-0.01	0.00	**0.014**
Pre-existing chronic pain	[*vs.* no]	0.28	0.18	0.38	**<0.001**
Ward: any opioid	[*vs.* none]	0.02	-0.06	0.09	0.621
Income level: high	[*vs.* others]	0.27	0.05	0.49	**0.026**

Estimated marginal means (covariate adjusted means) and 95% confidence intervals of the **pain composite side effects sub-score** (PCS_{side}, n = 4,982 patients). Results are shown for the peri-operative treatment protocol groups. The PCS_{side} was z-standardized before modelling.

group	mean	95% CI	
Incomplete	0.06	-0.08	0.20
Intra-operative	-0.10	-0.30	0.09
Post-operative	0.02	-0.13	0.17
Full care	-0.17	-0.33	-0.01

Contrasts (differences of the estimated marginal means) and 95% confidence intervals for the **pain composite side effects sub-score** (PCS_{side}, n = 4,982 patients). Significant contrasts ($p < 0.05$) are printed in bold. The PCS_{side} was z-standardized before modelling. Thus, differences can be interpreted as differences in standard deviations.

contrast			estimate	95% CI		p
Incomplete	*vs.*	Intra-operative	-0.17	-0.32	-0.01	0.152
Incomplete	*vs.*	Post-operative	-0.04	-0.16	0.07	0.815
Incomplete	*vs.*	Full care	-0.23	-0.37	-0.10	**0.003**
Intra-operative	*vs.*	Post-operative	0.12	-0.05	0.29	0.466
Intra-operative	*vs.*	Full care	-0.07	-0.23	0.09	0.815
Post-operative	*vs.*	Full care	-0.19	-0.31	-0.07	**0.007**

3 Sensitivity analysis for the total Pain Composite Score

Sensitivity analysis excluding one hospital with a high patient number. Results of the multi-level regression models using the pain composite score (0-10) as dependent variable. Regression coefficients (β) including 95% confidence intervals (95%CI) and p-values (p) are shown. In addition, results for the patient sample with regional anaesthesia are shown. Significant p-values are printed in bold.

		total cohort [n = 3 041]				regional anaesthesia [n = 2 397]			
**variable**	**reference**	**β**	**95% CI**		**p**	**β**	**95% CI**		**p**
**(intercept)**		-0.16	-0.55	0.22	0.419	-0.15	-0.62	0.31	0.527
**Peri-operative care group:**									
Intra-operative [only]	[*vs.* Incomplete]	-0.24	-0.43	-0.06	**0.009**	-0.22	-0.42	-0.02	**0.028**
Post-operative [only]	[*vs.* Incomplete]	-0.09	-0.20	0.03	0.154	-0.01	-0.14	0.13	0.917
Full care	[*vs.* Incomplete]	-0.38	-0.52	-0.25	**< 0.001**	-0.39	-0.54	-0.24	**< 0.001**
**group:**									
General anaesthesia	[*vs.* regional anaesthesia]	0.28	0.13	0.42	**< 0.001**		*not modelled*		
Combined RA & GA	[*vs.* regional anaesthesia]	0.16	-0.08	0.39	0.196				
**Age**	[years]	-0.01	-0.01	0.00	**0.030**	-0.01	-0.02	0.00	**0.032**
**Pre-existing chronic pain: yes**	[*vs.* no]	0.27	0.15	0.39	**< 0.001**	0.23	0.10	0.36	**< 0.001**
**Intra-operative: neuraxial morphine**	[vs. others/none]		*not modelled*			-0.12	-0.23	-0.01	**0.032**
**Ward: opioid**	[*vs.* no]	0.18	0.10	0.26	**< 0.001**	0.23	0.14	0.32	**< 0.001**
**Income level: high**	[*vs.* others]	0.50	0.06	0.93	**0.037**	0.49	0.00	0.99	0.068

Estimated marginal means (EMM, z-scale) including 95% confidence intervals (CI) for the different multi-level models and models from sensitivity analysis. Pain composite scores served as dependent variables and covariates are listed at the end of the table.

**Model I: total sample [n = 4 982]**			
**Care group**	**EMM[1]**	**95%CI**	
Incomplete	0.04	-0.17	0.25
intra-operative (only)	-0.23	-0.47	0.02
PACU/normal-ward (only)	-0.03	-0.25	0.18
Full Care	-0.32	-0.54	-0.09
**Model II: without one hospital [n = 3 041]**			
**Care group**	**EMM[1]**	**95%CI**	
Incomplete	0.22	-0.03	0.46
intra-operative (only)	-0.03	-0.31	0.26
PACU/normal-ward (only)	0.13	-0.11	0.38
Full Care	-0.17	-0.42	0.09
**Model III: regional anaesthesia [n = 4 213]**			
**Care group**	**EMM[2]**	**95%CI**	
Incomplete	-0.13	-0.35	0.10
intra-operative (only)	-0.36	-0.62	-0.11
PACU/normal-ward (only)	-0.14	-0.37	0.09
Full Care	-0.49	-0.73	-0.26
**Model IV: regional anaesthesia/without one hospital [n = 2 397]**			
**Care group**	**EMM[2]**	**95%CI**	
Incomplete	-0.01	-0.29	0.27
intra-operative (only)	-0.23	-0.55	0.08
PACU/normal-ward (only)	-0.02	-0.30	0.26
Full Care	-0.40	-0.69	-0.12

[1] **adjusted for:** protocol of anaesthesia (regional anaesthesia *vs.* general anaesthesia *vs.* mixed protocols), chronic pre-existing pain (yes vs. no), age (years), opioid administration on the ward (yes vs. no), income level of the country (high vs. others), intra-operative use of dexamethasone (vs. no)

[2] **adjusted for:** chronic pre-existing pain (yes vs. no), age (years), opioid administration on the ward (yes vs. no), income level of the country (high vs. others), intra-operative use of neuraxial morphine (vs. no) and dexamethasone (vs. no)

Contrasts of the different multi-level models and multi-level models for sensitivity analysis for different sub-groups of peri-operative treatment protocol. Pain composite scores served as dependent variables. P-values were adjusted using the Bonferroni-Holm method. Significant p-values ($p < 0.05$) are printed in bold.

		**Model**			
		**I**	**II**	**III**	**IV**
**contrast**		**p**	**p**	**p**	**p**
**Incomplete *vs.***	**Intra-operative**	**0.002**	**0.037**	**0.021**	0.114
**Incomplete *vs***	**Post-operative**	0.412	0.263	0.837	0.917
**Incomplete *vs***	**Full care**	**< 0.001**	**< 0.001**	**< 0.001**	**< 0.001**
**Intra-operative *vs.***	**Post-operative**	0.065	0.263	**0.045**	0.114
**Intra-operative *vs.***	**Full care**	0.412	0.263	0.261	0.121
**Post-operative *vs.***	**Full care**	**< 0.001**	**< 0.001**	**< 0.001**	**< 0.001**

4 Model details: Regional anaesthesia

4.1 Pain Composite Score: total

Model parameter for the **total pain composite score** ($PCS_{:totall}$, n = 4,213 patients). The PCS_{total} was z-standardized before modelling. Significant regression coefficients are printed in bold (coefficient of determination: $R^2_{marginal}$= 0.035, $R^2_{conditional}$= 0.236).

Variable	[Reference/Unit]	β	95%CI		p
(Intercept)		-0.30	-0.66	0.07	0.125
**Perioperative treatment protocol:**					
Intra-operative	[*vs.* Incomplete]	-0.24	-0.40	-0.07	**0.005**
Post-operative	[*vs.* Incomplete]	-0.01	-0.15	0.12	0.837
Full care	[*vs.* Incomplete]	-0.36	-0.51	-0.22	**<0.001**
Age	[years]	0.00	-0.01	0.00	0.266
Pre-existing chronic pain	[*vs.* no]	0.17	0.07	0.28	**0.002**
Intra-operative neuraxial morphine: yes	[*vs.* no]	-0.16	-0.27	-0.05	**0.004**
Ward: any opioid	[*vs.* none]	0.20	0.12	0.28	**<0.001**
Income level: high	[*vs.* others]	0.31	-0.11	0.73	0.162

Estimated marginal means (covariate adjusted means) and 95% confidence intervals of the **total pain composite score** (n = 4,213 patients). Results are shown for the peri-operative treatment protocol groups. The PCS_{total} was z-standardized before modelling.

group	mean	95% CI	
Incomplete	-0.13	-0.35	0.10
Intra-operative	-0.36	-0.62	-0.11
Post-operative	-0.14	-0.37	0.09
Full care	-0.49	-0.73	-0.26

Contrasts (differences of the estimated marginal means) and 95% confidence intervals for the **total pain composite score** (PCS_{total}, n = 4,213 patients). Significant contrasts ($p < 0.05$) are printed in bold. The PCS_{total} was z-standardized before modelling. Thus, differences can be interpreted as differences in standard deviations.

contrast			estimate	95% CI		p
Incomplete	*vs.*	Intra-operative	-0.24	-0.40	-0.07	**0.021**
Incomplete	*vs.*	Post-operative	-0.01	-0.15	0.12	0.837
Incomplete	*vs.*	Full care	-0.36	-0.51	-0.22	**0.000**
Intra-operative	*vs.*	Post-operative	0.22	0.04	0.40	**0.045**
Intra-operative	*vs.*	Full care	-0.13	-0.29	0.04	0.261
Post-operative	*vs.*	Full care	-0.35	-0.47	-0.22	**0.000**

4.2 Pain Composite Score: intensity sub-score

Model parameter for the **pain composite intensity sub-score** ($PCS_{intensity}$, n = 4,213 patients). The $PCS_{intensity}$ was z-standardized before modelling. Significant regression coefficients are printed in bold (coefficient of determination: $R^2_{marginal}$= 0.057, $R^2_{conditional}$= 0.295).

Variable	[Reference/Unit]	β	95%CI		p
(Intercept)		-0.47	-0.85	-0.09	**0.023**
**Perioperative treatment protocol:**					
Intra-operative	[*vs.* Incomplete]	-0.13	-0.28	0.03	0.109
Post-operative	[*vs.* Incomplete]	-0.01	-0.13	0.12	0.926
Full care	[*vs.* Incomplete]	-0.15	-0.29	-0.02	**0.029**
Age	[years]	0.01	0.00	0.01	**0.049**
Pre-existing chronic pain	[*vs.* no]	0.11	0.01	0.21	**0.029**
Intra-operative neuraxial morphine: yes	[*vs.* no]	-0.33	-0.43	-0.23	**<0.001**
Ward: any opioid	[*vs.* none]	0.22	0.14	0.30	**<0.001**
Income level: high	[*vs.* others]	-0.04	-0.49	0.40	0.847

Estimated marginal means (covariate adjusted means) and 95% confidence intervals of the **pain composite intensity sub-score** ($PCS_{intensity}$, n = 4,213 patients). Results are shown for the peri-operative treatment protocol groups. The $PCS_{intensity}$ was z-standardized before modelling.

group	mean	95% CI	
Incomplete	-0.33	-0.56	-0.09
Intra-operative	-0.46	-0.72	-0.19
Post-operative	-0.33	-0.58	-0.09
Full care	-0.48	-0.73	-0.24

Contrasts (differences of the estimated marginal means) and 95% confidence intervals for the **pain composite intensity sub-score** ($PCS_{intensity}$, n = 4,213 patients). Significant contrasts ($p < 0.05$) are printed in bold. The $PCS_{intensity}$ was z-standardized before modelling. Thus, differences can be interpreted as differences in standard deviations.

contrast			estimate	95% CI		p
Incomplete	*vs.*	Intra-operative	-0.13	-0.28	0.03	0.435
Incomplete	*vs.*	Post-operative	-0.01	-0.13	0.12	0.999
Incomplete	*vs.*	Full care	-0.15	-0.29	-0.02	0.145
Intra-operative	*vs.*	Post-operative	0.12	-0.05	0.29	0.473
Intra-operative	*vs.*	Full care	-0.02	-0.18	0.13	0.999
Post-operative	*vs.*	Full care	-0.15	-0.27	-0.03	0.094

4.3 Pain Composite Score: interference sub-score

Model parameter for the **pain composite interference sub-score** ($PCS_{interference}$, n = 4,213 patients). The $PCS_{interference}$ was z-standardized before modelling. Significant regression coefficients are printed in bold (coefficient of determination: $R^2_{marginal}$= 0.05, $R^2_{conditional}$= 0.242).

Variable	[Reference/Unit]	β	95%CI		p
(Intercept)		-0.22	-0.58	0.15	0.254
**Perioperative treatment protocol:**					
Intra-operative	[*vs.* Incomplete]	-0.17	-0.34	-0.01	**0.042**
Post-operative	[*vs.* Incomplete]	-0.03	-0.17	0.10	0.647
Full care	[*vs.* Incomplete]	-0.34	-0.49	-0.20	**<0.001**
Age	[years]	0.00	-0.01	0.00	0.456
Pre-existing chronic pain	[*vs.* no]	0.07	-0.04	0.17	0.215
Intra-operative neuraxial morphine: yes	[*vs.* no]	-0.37	-0.48	-0.26	**<0.001**
Ward: any opioid	[*vs.* none]	0.18	0.10	0.26	**<0.001**
Income level: high	[*vs.* others]	0.41	-0.01	0.83	0.068

Estimated marginal means (covariate adjusted means) and 95% confidence intervals of the **pain composite interference sub-score** ($PCS_{interference}$, n = 4,213 patients). Results are shown for the peri-operative treatment protocol groups. The $PCS_{interference}$ was z-standardized before modelling.

group	mean	95% CI	
Incomplete	-0.14	-0.36	0.09
Intra-operative	-0.31	-0.57	-0.05
Post-operative	-0.17	-0.40	0.06
Full care	-0.48	-0.71	-0.25

Contrasts (differences of the estimated marginal means) and 95% confidence intervals for the **pain composite interference sub-score** ($PCS_{interference}$, n = 4,213 patients). Significant contrasts ($p < 0.05$) are printed in bold. The $PCS_{interference}$ was z-standardized before modelling. Thus, differences can be interpreted as differences in standard deviations.

contrast			estimate	95% CI		p
Incomplete	*vs.*	Intra-operative	-0.17	-0.34	-0.01	0.166
Incomplete	*vs.*	Post-operative	-0.03	-0.17	0.10	0.647
Incomplete	*vs.*	Full care	-0.34	-0.49	-0.20	**<0.001**
Intra-operative	*vs.*	Post-operative	0.14	-0.04	0.32	0.247
Intra-operative	*vs.*	Full care	-0.17	-0.34	0.00	0.166
Post-operative	*vs.*	Full care	-0.31	-0.44	-0.19	**<0.001**

4.4 Pain Composite Score: side effects sub-score

Model parameter for the **pain composite side effects sub-score** (PCS_{side}, n = 4,213 patients). The PCS_{side} was z-standardized before modelling. Significant regression coefficients are printed in bold (coefficient of determination: $R^2_{marginal}$= 0.035, $R^2_{conditional}$= 0.083).

Variable	[Reference/Unit]	β	95%CI		p
(Intercept)		-0.06	-0.30	0.19	0.643
**Perioperative treatment protocol:**					
Intra-operative	[*vs.* Incomplete]	-0.23	-0.40	-0.06	**0.008**
Post-operative	[*vs.* Incomplete]	0.02	-0.11	0.15	0.771
Full care	[*vs.* Incomplete]	-0.27	-0.41	-0.13	**<0.001**
Age	[years]	-0.01	-0.01	0.00	**0.003**
Pre-existing chronic pain	[*vs.* no]	0.25	0.14	0.36	**<0.001**
Intra-operative neuraxial morphine: yes	[*vs.* no]	0.36	0.26	0.47	**<0.001**
Ward: any opioid	[*vs.* none]	0.07	-0.01	0.16	0.084
Income level: high	[*vs.* others]	0.20	-0.01	0.42	0.081

Estimated marginal means (covariate adjusted means) and 95% confidence intervals of the **pain composite side effects sub-score** (PCS_{side}, n = 4,213 patients). Results are shown for the peri-operative treatment protocol groups. The PCS_{side} was z-standardized before modelling.

group	mean	95% CI	
Incomplete	0.12	-0.01	0.26
Intra-operative	-0.10	-0.29	0.08
Post-operative	0.14	0.00	0.29
Full care	-0.15	-0.30	0.00

Contrasts (differences of the estimated marginal means) and 95% confidence intervals for the **pain composite side effects sub-score** (PCS_{side}, n = 4,213 patients). Significant contrasts ($p < 0.05$) are printed in bold. The PCS_{side} was z-standardized before modelling. Thus, differences can be interpreted as differences in standard deviations.

contrast			estimate	95% CI		p
Incomplete	*vs.*	Intra-operative	-0.23	-0.40	-0.06	**0.031**
Incomplete	*vs.*	Post-operative	0.02	-0.11	0.15	0.999
Incomplete	*vs.*	Full care	-0.27	-0.41	-0.13	**<0.001**
Intra-operative	*vs.*	Post-operative	0.25	0.06	0.43	**0.031**
Intra-operative	*vs.*	Full care	-0.04	-0.21	0.12	0.999
Post-operative	*vs.*	Full care	-0.29	-0.42	-0.17	**<0.001**
